# The wellbeing of health care workers

**DOI:** 10.1080/02813432.2021.2012352

**Published:** 2021-12-24

**Authors:** Emil L. Sigurdsson

**Affiliations:** Department of Family Medicine, University of Iceland Head of the Development Centre for Primary Healthcare in Iceland, Alfabakki 16109 Reykjavik, Iceland

The wellbeing of doctors and other health care workers (HCWs) is extremely important for the health care service. Stress and work overload definitely affect the quality of care for patients and also influence the health and wellbeing of HCWs. Studies have convincingly shown that the wellbeing of HCWs improves the quality of care, productivity and patient satisfaction [1]. Furthermore, the likelihood of making a major medical error is approximately 50% higher among doctors with high levels of burnout [2]. Another aspect of stress and work-overload is the difficulties of recruiting and retaining general practitioners (GPs) and professional exhaustion and demoralization are among the factors that are linked to those problems [3].

The eighth National GP Worklife Survey on job satisfaction among GPs in England showed the highest levels of stress since the survey began in 1998. Data from the survey published in 2017 revealed that 35% of GPs were intending to quit direct patient care within the next five years [4]. In Scotland, 26% of GPs said they are unlikely to be working in general practice in five years’ time, due to workloads and unmanageable stress [5]. In an unpublished survey done among HCWs at the largest primary health care center in Iceland, 10% of the employees claims that they often think about not coming back to work the following day. Prior to Covid-19 approximately one-third of UK doctors suffered from burnout and secondary traumatic stress [1].

The Covid-19 pandemic has led to even more workload and new tasks had to be accepted [6]. In a newly published paper on the determinants of burnout and other aspects of psychological well-being in HCWs during the Covid-19 pandemic, several important findings were reported [6]. Predictors of burnout, anxiety and depression were found to be patient-facing role or being a nurse. However, factors inversely correlated with these diseases included being tested for SARS-CoV-2, safety attitudes, job role and gender. Female gender was predictive for anxiety but was found to be inversely correlated with depression. The significant burden of burnout, anxiety and depression was found amongst HCWs. The proportion of respondents at a high risk of burnout was 67% and 20% met the criteria for anxiety and 11% for depression. Clinical roles confer a higher risk of burnout than non-clinical works. The fear of becoming infected and the burden of having to wear a piece of personal protective equipment are among the factors that influence the well-being of HCWs.

In 2019 the General Medical Council in the UK published a report, caring for doctors caring for patients [7]. In that report it is stated that to ensure wellbeing and motivation at work and to minimize workplace stress, people have three core needs and that all of these three cores must be met:

- Autonomy/control – the need to have control over our work lives, and to act consistently with our work and life values.
- Belonging – the need to be connected to, cared for, and caring of others around us in the workplace and to feel valued, respected and supported.
- Competence – the need to experience effectiveness and deliver valued outcomes, such as high-quality care.

Furthermore, the report points out ways that can be used to transform doctors’ workplaces, so they thrive and flourish and are better able to provide the compassionate and high-quality care they want to deliver. The quality of care and improvements in the quality of care are extremely difficult if the HCWs wellbeing is not acceptable.

WHO has designated 2021 as the International year of Health and Care Workers [8] and despite the fact that the wellbeing of HCWs has been a concern for decades the Covid-19 has drawn more attention to the importance of the wellbeing of clinicians [9]. It must be ensured that the work lives of HCWs are both fulfilling and attractive. In that way, we enhance the possibilities of providing a good solid working environment so that they can deliver their patients a quality health care service that is both sustainable and beneficial for HCWs. It is imperative to implement this so that the health care service operates in an acceptable and beneficial way. Complacency is not an option when confronting the well-being of HCWs.
